# Antimicrobial Resistance in ESKAPE Pathogens: A Retrospective Epidemiological Study at the University Hospital of Palermo, Italy

**DOI:** 10.3390/antibiotics14020186

**Published:** 2025-02-12

**Authors:** Luca Pipitò, Raffaella Rubino, Giulio D’Agati, Eleonora Bono, Chiara Vincenza Mazzola, Sofia Urso, Giuseppe Zinna, Salvatore Antonino Distefano, Alberto Firenze, Celestino Bonura, Giovanni M. Giammanco, Antonio Cascio

**Affiliations:** 1 Department of Health Promotion, Mother and Child Care, Internal Medicine and Medical Specialties “G D’Alessandro”, University of Palermo, 90133 Palermo, Italy; giulio.dagati@community.unipa.it (G.D.); eleonora.bono@community.unipa.it (E.B.); chiaravincenza.mazzola@community.unipa.it (C.V.M.); sofia.urso@community.unipa.it (S.U.); alberto.firenze@unipa.it (A.F.); celestino.bonura@unipa.it (C.B.); giovanni.giammanco@unipa.it (G.M.G.); 2 Infectious and Tropical Disease Unit, Sicilian Regional Reference Center for the Fight Against AIDS, AOU Policlinico “P. Giaccone”, 90127 Palermo, Italy; raffaella.rubino@policlinico.pa.it; 3 Antimicrobial Stewardship Team, AOU Policlinico “P. Giaccone”, 90127 Palermo, Italy; salvatoreantonino.distefano@policlinico.pa.it; 4 Department of Surgery, Dentistry, Paediatrics, and Gynaecology, Division of Cardiac Surgery, University of Verona Medical School, 37129 Verona, Italy; giuseppe.zinna@studenti.univr.it; 5 Microbiology and Virology Unit, AOU Policlinico “P. Giaccone”, 90127 Palermo, Italy

**Keywords:** ESKAPE, antimicrobial resistance, hospital epidemiology bacteria, *Enterococcus faecium*, *Staphylococcus aureus*, *Klebsiella pneumoniae*, *Acinetobacter baumannii*, *Pseudomonas aeruginosa*, *Enterobacter* spp.

## Abstract

Background: Antimicrobial resistance (AMR) is an escalating global health threat, projected to cause over 40 million deaths by 2050. ESKAPE pathogens (*Enterococcus faecium*, *Staphylococcus aureus*, *Klebsiella pneumoniae*, *Acinetobacter baumannii*, *Pseudomonas aeruginosa*, and *Enterobacter* spp.) are major contributors to nosocomial infections and AMR. We evaluated the epidemiology and AMR prevalence of ESKAPE pathogens at the University Hospital in Palermo between January 2018 and July 2023, analyzing factors associated with mortality in patients with positive blood cultures. Methods: Microbiological data from all specimen types were collected using the Business Intelligence system Biwer, excluding duplicates. We assessed the prevalence and trends of ESKAPE isolates and AMR over time. Clinical data from hospital discharge forms were used to evaluate factors associated with mortality in patients with ESKAPE-positive blood cultures. Differences in AMR prevalence between blood and non-blood isolates were examined. Results: A total of 11,607 specimens from 4916 patients were analyzed. Most patients were admitted to Internal Medicine (19.4%), the ICU (13.2%), and General Surgery (9.9%). Additionally, 21.5% of the specimens were collected from ICU-admitted patients. Blood cultures accounted for 14.3% of the specimens, urine for 25.3%, respiratory secretions for 22.1%, and skin and mucosal swabs for 20.9%. The prevalence of all isolates increased progressively, peaking in 2021. The vancomycin-resistant *E. faecium* prevalence was 19.4%, with a significant upward trend, while oxacillin-resistant *S. aureus* prevalence was 35.0%, showing a significant decline. *A. baumannii* exhibited high resistance to all antibiotics tested except for colistin and cefiderocol. Carbapenemase resistance was 55.0% in *K. pneumoniae*, 20.4% in *P. aeruginosa*, and 4.6% in *Enterobacter* spp. *P. aeruginosa* showed a significant decrease in meropenem resistance. *K. pneumoniae* and *A. baumannii* bloodstream infections were linked to higher mortality risk.

## 1. Introduction

Antimicrobial resistance (AMR) is considered a silent pandemic, projected to cause over 40 million deaths by 2050 [1]. According to WHO data, AMR was directly responsible for approximately 1.27 million deaths worldwide in 2019 and contributed to a total of 4.95 million deaths [2]. The increasing prevalence of antimicrobial-resistant pathogens, particularly those acquired in hospital settings, poses a global threat and significantly burdens healthcare systems. The Infectious Diseases Society of America introduced the acronym ESKAPE to denote a group of nosocomial pathogens with a high capacity to develop AMR, including *Enterococcus faecium*, *Staphylococcus aureus*, *Klebsiella pneumoniae*, *Acinetobacter baumannii*, *Pseudomonas aeruginosa*, and *Enterobacter species*, responsible for most hospital-acquired infections [3]. The ESKAPE pathogens have been classified among the priority pathogens by the WHO [4]. Notably, based on disease burden, AMR, and the availability of effective antibiotics, *Acinetobacter baumannii* and Enterobacterales have been designated as critical priority pathogens. Meanwhile, *Enterococcus faecium*, *Pseudomonas aeruginosa*, and *Staphylococcus aureus* are categorized within the high-priority group [4]. The inappropriate use of antibiotics in humans and animals, the inconsistent application of disinfectants, globalization, hospital settings, and co-infections during the COVID-19 pandemic have all contributed to the spread of AMR, and ESKAPE pathogens have maintained an important role in nosocomial infections, even during the pandemic [5,6,7,8]. Nevertheless, to combat the rise in AMR, the WHO introduced the AWaRe (Access, Watch, Reserve) classification system to guide the appropriate use of antibiotics [9]. The Access group includes first- or second-line treatments for common infections, such as amoxicillin, doxycycline, and metronidazole. The Watch group consists of antibiotics for specific, limited indications due to their higher potential for resistance development, including ciprofloxacin, ceftriaxone, and azithromycin. The Reserve group encompasses last-line antibiotics reserved for confirmed or suspected multidrug-resistant (MDR) infections, such as linezolid, ceftobiprole, and meropenem-vaborbactam [9]. However, despite the introduction of several new antibiotics and antibiotic adjuvants, including novel β-lactamase inhibitors, ESKAPE pathogens still pose significant therapeutic challenges [10]. These organisms share key characteristics, including their ability to adapt and persist in modern healthcare settings, as well as a repertoire of intrinsic and acquired resistance mechanisms that have allowed them to emerge as major contributors to antimicrobial-resistant infections over time [10]. Furthermore, a recent study demonstrated that Gram-negative ESKAPE pathogens can develop antimicrobial resistance to new antibiotics not yet on the market, with 120 generations (60 days) of laboratory evolution sufficient for the bacterial strains to develop resistance [11]. Understanding epidemiology, resistance mechanisms, and potential therapeutic strategies against ESKAPE pathogens is crucial to mitigating their impact, informing public health policies, guiding antimicrobial stewardship programs, and ensuring the development of effective countermeasures. However, data on the epidemiology of ESKAPE pathogens in Italy and worldwide, including trends in antimicrobial resistance, remain limited and poorly documented. This study aims to evaluate ESKAPE pathogens’ epidemiology and AMR prevalence between 1 January 2018 and 6 July 2023 at the University Hospital in Palermo and assess factors associated with mortality in patients with bloodstream infections (BSIs).

## 2. Results

### 2.1. Specimens, Wards, and Isolates

**Figure 1:**
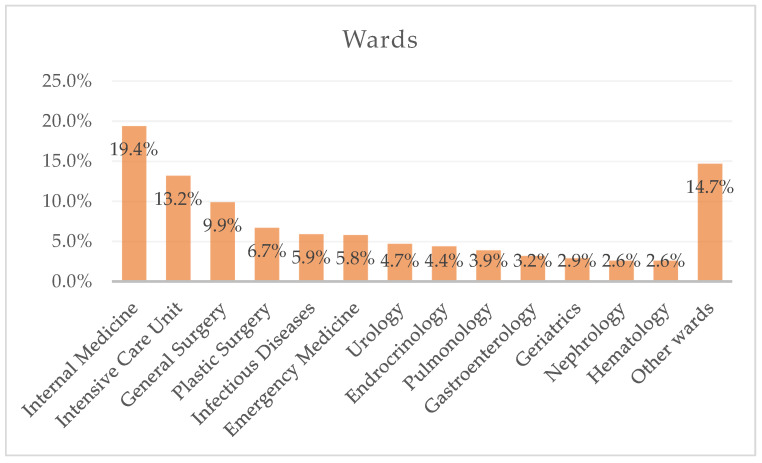
Admission wards of the 4916 patients at the time of specimen collection.

In our study, 11,607 specimens from 4916 patients were analyzed. The number of specimens associated with a single patient ranged from 1 to 45. The median age of the patients was 69 years (IQR 57–78). A total of 2770 patients were male (56.3%), and 58.4% of the specimens were collected from male patients. The wards of admission for these patients are depicted in Figure 1. Most patients were admitted to the Internal Medicine Unit (19.4%), the Intensive Care Unit (ICU, 13.2%), and the General Surgery Unit (9.9%). Only 5.9% were admitted to the Infectious Diseases Unit, while 14.7% were admitted to other wards.

**Figure 2:**
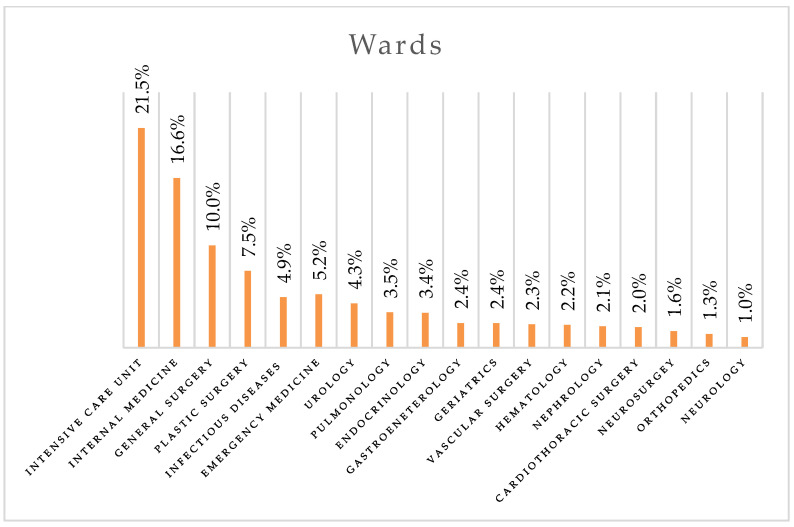
Distribution of collected specimens by ward.

The distribution of collected specimens according to wards is shown in Figure 2. Most of the specimens were from ICUs (21.5%), the Internal Medicine Unit (16.6%), and the General Surgery Unit (10.0%). A smaller proportion of specimens came from the Infectious Diseases Unit (4.9%) and other unspecified wards (6.9%).

**Figure 3:**
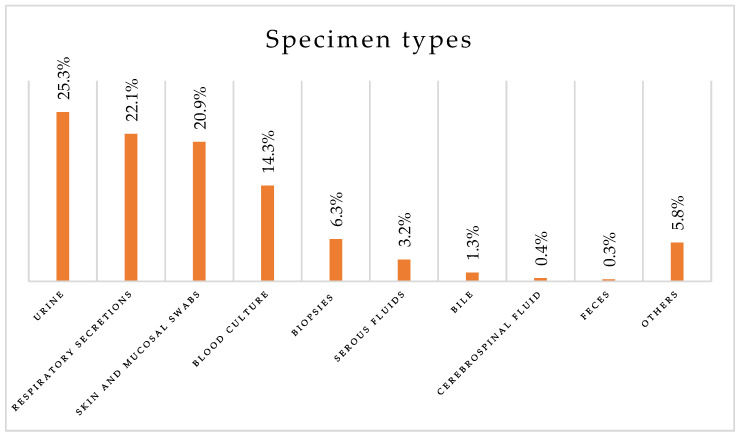
Types of biological specimens collected.

Most collected specimens were urine (25.3%) and respiratory secretions (22.1%), including sputum, bronchoalveolar lavage, and tracheal aspiration. Skin and mucosal swabs accounted for 20.9% of the specimens, while blood cultures constituted 14.3% (Figure 3).

**Figure 4:**
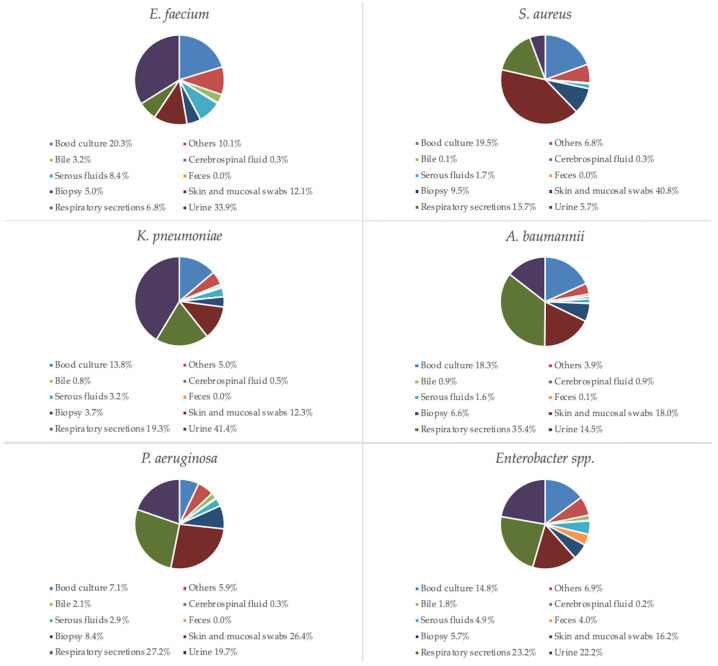
Percentage of specimen types by individual ESKAPE pathogen.

Regarding specimen type distribution for specific pathogen, most of *Enterococcus faecium* isolates were obtained from urine specimens (33.9%) and blood (20.3%), *Staphylococcus aureus* from mucocutaneous swabs (40.8%) and blood (19.5%), *Klebsiella pneumoniae* from urine (41.4%) and respiratory specimens (19.3%), *Acinetobacter baumannii* from respiratory specimens (35.4%) and blood (18.3%), *Pseudomonas aeruginosa* from respiratory specimens (27.2%) and mucocutaneous swabs (26.4%), and *Enterobacter* spp. from respiratory specimens (23.2%) and urine (22.2%), as shown in Figure 4. The *Enterobacter* spp. included *E. cloacae* (71.3%), *E. aerogenes* (17.4%), *E. hormaechei* (1.9%), *E. asburiae* (0.7%), *E. gergoviae* (0.6%), *E. kobei* (0.6%), *E. intermedius* (0.4%), *E. sakazakii* (0.1%), and other unspecified species (6.9%).

**Figure 5:**
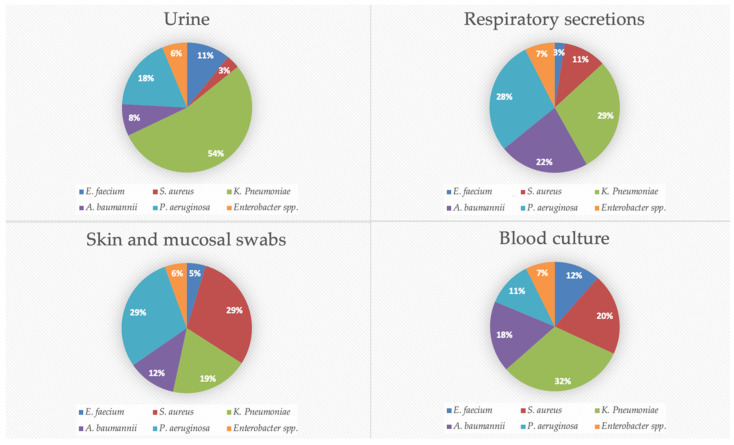
Distribution of isolates by specimen type.

**Figure 6:**
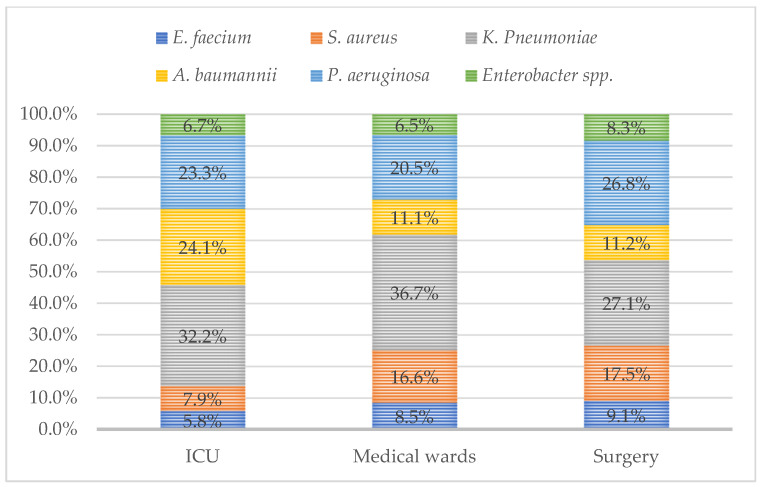
Prevalence of isolate types in ICUs, medical wards, and surgery units.

The prevalence of isolates according to main specimen type and unit of admission (ICUs vs. medical wards vs. surgery units) are depicted in Figure 5 and Figure 6, respectively. The main isolate from the ICU, medical wards, and surgery units was *K. pneumoniae*. A positive linear correlation was observed between *A. baumannii* isolation and ICU stay (r = 0.15422, *p* < 0.001).

### 2.2. Trends of Isolates

**Figure 7:**
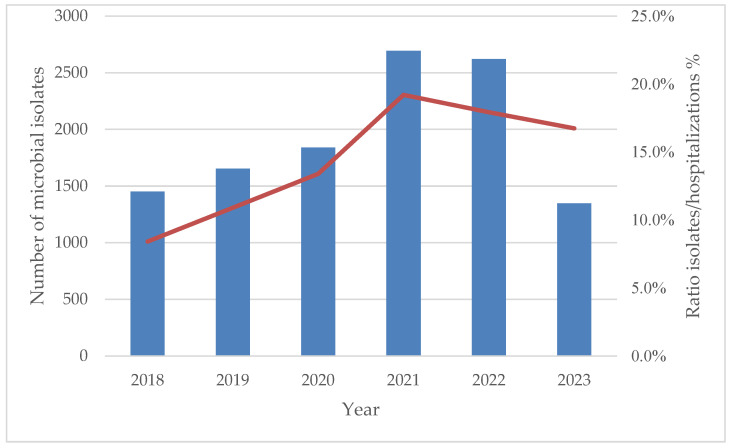
Absolute frequencies of microbiological isolates from 1 January 2018 to 6 July 2023 and ratios of isolates to admissions expressed as percentages.

The absolute frequency of isolates over the years, as well as the ratio of isolates to hospitalizations, showed a progressive increase, peaking in 2021 (19.2%), with a statistically significant trend (β = 0.86280; 95% CI: 0.00365, 0.03548; *p* = 0.02695), as depicted in Figure 7.

**Figure 8:**
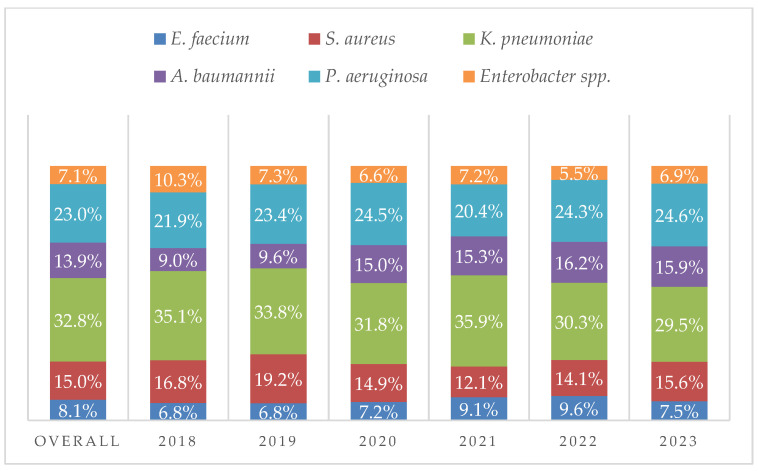
Percentages of microbiological isolates over the years.

The annual distribution of bacterial isolates (Figure 8) revealed a significant increase in the prevalence of *A. baumannii* (β = 0.89106; 95% CI: 0.00459, 0.02674; *p* = 0.01716). Statistically significant variations in the prevalence of *E. faecium*, *S. aureus*, *K. pneumoniae*, *P. aeruginosa*, and *Enterobacter* spp. isolations over the years of the study were not observed.

**Figure 9:**
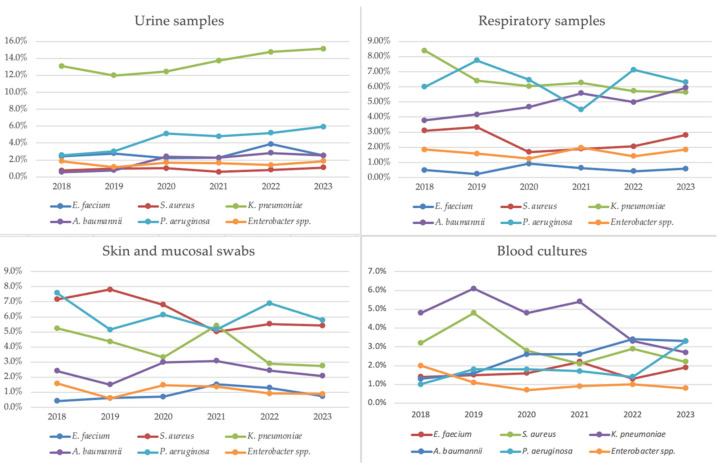
Trends of isolates per bacterium and specimen type relative to total collected specimens for each year, 2018–2023.

Significant trends were observed in the isolation of *A. baumannii*, with increases in blood cultures (β = 0.95519; 95% CI: 0.00251–0.00629; *p* = 0.00297), respiratory secretions (β = 0.92297; 95% CI: 0.00170–0.00636; *p* = 0.00867), and urine specimens (β = 0.87561; 95% CI: 0.00106–0.00799; *p* = 0.02225). For *K. pneumoniae*, there was an increase in isolates from urine specimens (β = 0.84536; 95% CI: 0.00070–0.01066; *p* = 0.03402) and a decrease from respiratory specimens (β = −0.82081; 95% CI: −0.00879 to −0.00015; *p* = 0.04529). Additionally, *P. aeruginosa* showed an increasing trend in urine specimens (β = 0.92296; 95% CI: 0.00278–0.01042; *p* = 0.00868), while *S. aureus* exhibited a decline in mucocutaneous swabs (β = −0.82685; 95% CI: −0.00963 to −0.00028; *p* = 0.04238). Trends in isolate distribution by specimen type are illustrated in Figure 9.

### 2.3. Antimicrobial Resistance Prevalence and Trends

Antimicrobial resistance data, stratified by bacterium and year, are presented in Table 1. The overall prevalence of vancomycin-resistant *E. faecium* (VRE) was 19.4%. A significantly increased vancomycin resistance was observed for *E. faecium* (β = 0.84903; 95% CI: 0.00717, 0.09825; *p* = 0.03247). The overall prevalence of methicillin-resistant *S. aureus* (MRSA) was 35.0%, and a reduction in oxacillin resistance for *S. aureus* (β = −0.94019; 95% CI: −28.92284, −86.76248; *p* = 0.00526) was reported. Furthermore, a decreasing trend for trimethoprim-sulfamethoxazole resistance (β = −0.90003; 95% CI: −0.02929, −0.00574; *p* = 0.01449) was noted. A consistently high resistance level was observed for *A. baumannii* to all antibiotics tested except colistin. The overall prevalence of carbapenemase-resistant *K. pneumoniae* was 55.0%. Although an increase in resistance prevalence was observed for *K. pneumoniae* to meropenem (*p* = 0.77108), meropenem-vaborbactam (*p* = 0.08432), and ceftazidime-avibactam (*p* = 0.79632), no statistically significant upward trends were identified. In contrast, *P. aeruginosa* showed a significant decreasing trend in resistance to meropenem (β = −0.92635; 95% CI: −0.03089, −0.00860; *p* = 0.00794) and ciprofloxacin (β = −0.95089; 95% CI: −0.04078, −0.01539; *p* = 0.00356). The overall prevalence of carbapenem resistance *P. aeruginosa* was 20.4%. For *Enterobacter* spp., significant decreases in resistance were noted for ciprofloxacin (β = −0.94668; 95% CI: −0.07737, −0.02772; *p* = 0.00419), cefepime (β = −0.97996; 95% CI: −0.05393, −0.03019; *p* = 0.00060), and gentamicin (β = −0.92351; 95% CI: −0.04356, −0.01170; *p* = 0.00855), and a low overall prevalence of carbapenem resistance was detected (4.6%). Additionally, a lower level of resistance to colistin was observed over the years across all Gram-negative bacteria. Cefiderocol was tested in 46 isolates: 37 *A. baumannii*, 4 *K. pneumoniae*, 4 *P. aeruginosa*, and 1 *E. cloacae*. Resistance was detected in two isolates, respectively, *A. baumannii* and *P. aeruginosa*.

### 2.4. In-Hospital Mortality and Multivariable Analysis for Patients with Blood Isolates

**Figure 10:**
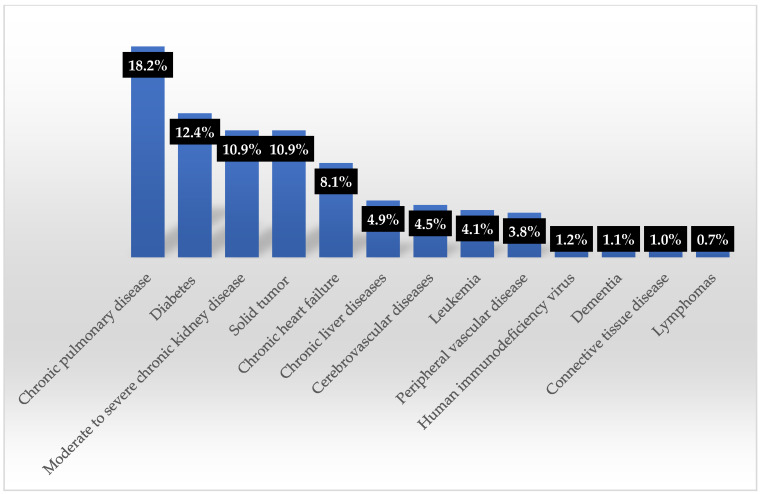
Comorbidities of patients with blood culture positive for ESKAPE pathogens.

Mortality and comorbidity data from H-SDFs were available for 81.3% of the bacteremia cases (1353 out of 1663). The main comorbidities of the patients are depicted in Figure 10. The median age was 68 years (IQR 56–76), with 61.3% of the specimens from male patients. The median CCI was 3 (IQR 2–5), and the in-hospital mortality rate was 38.1%.

Univariate analysis of in-hospital mortality revealed significant associations with *S. aureus* (cOR 0.33778; 95% CI: 0.247–0.463; *p* < 0.001), *K. pneumoniae* (cOR 1.46324; 95% CI: 1.158–1.849; *p* = 0.001), *A. baumannii* (cOR 2.18032; 95% CI: 1.643–2.894; *p* < 0.001), and *Enterobacter* spp. (cOR 0.37200; 95% CI: 0.222–0.622; *p* < 0.001). Multivariable analysis, adjusted for ICU admission (25.4%) and comorbidities using the CCI, demonstrated that *K. pneumoniae* and *A. baumannii* were independently associated with increased mortality risk (Table 2).

### 2.5. Profile of Antimicrobial Resistance, Blood Isolates vs. Others

The antimicrobial resistance profiles for the main antibiotics used in clinical practice were compared between isolates from blood and those from other specimen types. *K. pneumoniae* isolates from blood showed a higher prevalence of resistance to ceftazidime, piperacillin-tazobactam, meropenem, and ciprofloxacin (Table 3).

## 3. Methods

### 3.1. Data Collection

We conducted a single-center retrospective observational study. Microbiological data from all specimens collected at the University Hospital Paolo Giaccone in Palermo between 1 January 2018 and 6 July 2023 were retrieved from an institutional anonymized electronic microbiological information system. A total of 11,607 specimens from 4916 patients were collected. The specimens included blood, respiratory secretions, urine, biopsies, bile, cerebrospinal fluid, serous fluids, feces, skin and mucosal swabs, and others. Only isolates of ESKAPE pathogens were considered for inclusion in the study. Patient records included age, sex, bacterial isolates, hospital ward, and antimicrobial susceptibility patterns. Susceptibility test results were interpreted according to the European Committee on Antimicrobial Susceptibility Testing (EUCAST) criteria relevant to each year of the study period [12]. Resistance to ampicillin, vancomycin, teicoplanin, gentamicin, linezolid, and imipenem were reported for *E. faecium*. Resistance to oxacillin, vancomycin, teicoplanin, gentamicin, linezolid, daptomycin, trimethoprim-sulfamethoxazole, clindamycin, tetracycline, and moxifloxacin was reported for *S. aureus.* The Minimum Inhibitory Concentration test (MIC values > 2 mg/L) for oxacillin was used to determine methicillin resistance in *S. aureus* isolates. Resistance to amoxicillin-clavulanic acid, ceftazidime, piperacillin-tazobactam, cefepime, ceftolozane-tazobactam, meropenem, imipenem, ertapenem, ceftazidime-avibactam, meropenem-vaborbactam, trimetoprim-sulfamethoxazole, ciprofloxacin, gentamicin, and colistin was reported for *K. pneumoniae*. Resistance to meropenem, imipenem, trimethoprim-sulfamethoxazole, ciprofloxacin, gentamicin, and colistin was reported for *A. baumannii*. Resistance to ceftazidime, piperacillin-tazobactam, cefepime, ceftolozane-tazobactam, meropenem, imipenem, ceftazidime-avibactam, ciprofloxacin, tobramycin, and colistin was reported for *P. aeruginosa*. Resistance to ciprofloxacin, colistin, cefepime, amoxicillin-clavulanic acid, gentamicin, ertapenem, imipenem, meropenem, trimetoprim-sulfamethoxazole, ceftazidime, ceftazidime-avibactam, ceftolozane-tazobactam, meropenem-vaborbactam, and piperacillin-tazobactam was reported for *Enterobacter* spp. In addition, cefiderocol was tested in 46 cases of Gram-negative ESKAPE isolates. Phenotypic resistance to meropenem and/or imipenem was used to determine the prevalence of carbapenem resistance. The data used in this study were collected through a Business Intelligence system, Biwer, developed by Werfen [13]. By leveraging a native integration with the microbiology laboratory’s information system, advanced data acquisition was achieved. The same microbial isolate from the same specimen type of the same patient, collected within 7 days, was considered a duplicate and, therefore, excluded from the final extracted data. Multiple specimens could be included for each patient. We reported the prevalence of ESKAPE pathogens and antimicrobial resistance to the main antibiotics used in clinical practice annually, and trends were assessed over time. Wards of admission were classified into medical wards, ICUs, and surgical wards, and isolate types were categorized according to that classification. The correlation between isolate type and ward was evaluated.

### 3.2. Microbiological Specimens

Identification of the microorganisms isolated from clinical specimens was routinely performed using matrix-assisted laser desorption ionization-time of flight mass spectrometry (MALDI Biotyper, Bruker Daltonics, Billerica, MA, USA). Antimicrobial susceptibility testing (AST) for antibiotics other than cefiderocol was performed using an automated system (Phoenix, Becton Dickinson Diagnostics, Sparks, MD, USA). Cefiderocol AST was performed through a gradient test (Liofilchem S.r.L., Roseto degli Abruzzi, Italy) with a cefiderocol concentration range of 0.016–256 mg/L. MICs were interpreted according to the EUCAST for the interested year [12].

### 3.3. Patients with Positive Blood Culture

For patients with positive blood cultures, data from hospital discharge forms (H-SDFs), including comorbidities and vital status at discharge, were retrospectively collected and subsequently analyzed. Up to six diagnoses can be included in a patient’s H-SDF. Charlson comorbidity index (CCI) was calculated using diagnoses from H-SDFs and the patient’s age. CCI score includes age, history of myocardial infection, chronic heart failure, peripheral vascular disease, history of a cerebrovascular accident, dementia, chronic pulmonary disease, connective tissue disease, peptic ulcer disease, liver disease, diabetes mellitus, hemiplegia, moderate to severe chronic kidney disease, solid tumor, leukemia, lymphoma, and acquired immunodeficiency syndrome. Microorganisms from blood isolates associated with higher mortality in these patients were evaluated using univariate analysis, and the results were adjusted for CCI in a multivariable analysis. Lastly, it was determined that the prevalence of antimicrobial resistance was different between blood isolates and isolates from other specimen types. As the data collection system is anonymized, in accordance with the Italian Data Protection Authority, neither ethical committee approval nor informed consent was required. Regional health authorities routinely use anonymized data for both epidemiological and administrative purposes.

### 3.4. Statistical Analysis

Data are presented as numbers and percentages for categorical variables, and the continuous data are expressed as the median and interquartile range (IQR). Differences in medians were evaluated using the Mann-Whitney U test, and the χ^2^ test was applied to categorical variables. Statistical significance was set at a *p*-value < 0.05. Annual trends for the percentage of ESKAPE microbiological isolates were investigated using a linear regression model and estimates of unstandardized coefficients (β) and their confidence interval (95% CI). Pearson’s correlation coefficient was computed to verify the existence of correlations among bacteria isolate types and wards. Crude odds ratios (cORs) and their 95% CI for the association between mortality and potential risk factors were calculated using univariate analysis. The adjusted OR (aOR) was calculated using logistic regression analysis to identify the factors independently associated with mortality. The logistic regression analysis included only factors associated with mortality in the univariate analysis. For the statistical analysis, IBM SPSS, version 26 was used.

## 4. Discussion

ESKAPE pathogens represent a significant global health concern due to their ability to develop antibiotic resistance and cause severe infections in hospitalized patients with predisposing factors [10]. These pathogens are associated with increased hospital length of stay, higher healthcare costs, and a rise in mortality rates [14]. AMR is not confined to developed countries. Both high-income and low- and middle-income countries are facing a rising prevalence of AMR among ESKAPE pathogens [15]. Our study examined the frequency and antimicrobial resistance profiles of ESKAPE pathogens across various specimen types. Most of the collected specimens were urine and respiratory secretions, while blood cultures accounted for 14.3% of the total. Most samples were obtained from ICUs, where patients are at a higher risk of developing opportunistic infections. Skin and mucosal swabs were the third most common type of specimen; however, the diagnostic value of mucocutaneous swabs is sometimes limited, as the detection of these bacteria does not necessarily indicate infection but may instead reflect colonization. Nevertheless, data on the prevalence of ESKAPE pathogens in these samples provide valuable insights into the local epidemiology of circulating bacteria. Regarding specific pathogens, *E. faecium* was primarily isolated from urine and blood cultures, *S. aureus* from skin and mucosal swabs as well as blood cultures, *K. pneumoniae* from urine and respiratory specimens, *A. baumannii* from respiratory specimens and blood cultures, *P. aeruginosa* from respiratory specimens and skin and mucosal swabs, and *Enterobacter* spp. from respiratory specimens and urine. Notably, *K. pneumoniae* was the most frequently isolated pathogen in urine, respiratory secretions, and blood cultures. Variations in pathogen distribution were observed across different specimen types and hospital wards. *A. baumannii* was predominantly found in ICUs, while *S. aureus* showed a slightly higher prevalence in surgical units. Furthermore, *A. baumannii* isolation was correlated with ICU admission, with the highest prevalence observed in respiratory secretions. Additionally, a significant increase in *A. baumannii* detection in blood cultures was noted between 2018 and 2022. For *K. pneumoniae*, a decreasing trend in isolation from urine and respiratory specimens was observed. However, it remained the most frequently isolated pathogen in ICUs, medical wards, and surgical units. The prevalence of ESKAPE isolates increased over the study period, peaking in 2021 during the COVID-19 pandemic and showing a moderate decrease after. The pandemic exacerbated the issue of AMR, highlighting the problem of antibiotic resistance driven by the overuse of antibiotics, antimicrobial soaps, disinfectant cleaners, and secondary infections in COVID-19 patients. Reports from different regions worldwide have documented an increase in microorganisms with limited treatment options, such as *A. baumannii* and *E. faecium* [16]. A review published by Lai et al. in March 2021 summarized data on antimicrobial resistance during the initial year of the COVID-19 pandemic. The review reported a rapid increase in MDR organisms, including extended-spectrum β-lactamase (ESBL)-producing *K. pneumoniae*, carbapenem-resistant New Delhi metallo-β-lactamase-producing *Enterobacterales*, *A. baumannii*, and MRSA in ICUs across China, France, India, the United States, Taiwan, and other regions [17]. An Italian monocentric study revealed a higher prevalence of ESKAPE pathogens in COVID-19 patients compared to non-COVID-19 patients, with *Acinetobacter baumannii* being the predominant isolate (58.7%) among COVID-19 patients. This pathogen was the leading cause of bloodstream infections in this group and was associated with the highest mortality rate (68.7%). Notably, the rise in MDR organisms appeared to be particularly linked to COVID-19 in ICU settings [18]. Moreover, a study conducted in Romania reported a high prevalence of *vanA*-positive *E. faecium* colonization and MDR organisms among COVID-19 patients during the pandemic, with several cases of VRE species being identified [19].

According to AMR, we found an increasing prevalence of VRE, for which the remaining therapeutic options are minimal, generally restricted to linezolid, daptomycin, and newer-generation tetracycline antibiotics [10]. Oritavancin has shown in vitro effectiveness on *van-A* strains, but clinical data are severely lacking [20]. Our study highlighted a significant increasing trend in VRE isolates, with an overall prevalence of 19.4%. However, Italian Istituto Superiore di Sanità (ISS) data showed a prevalence of vancomycin resistance of 30.7% for Sicily (Italy) in 2023 [21]. For *S. aureus*, we observed a reduction in MRSA prevalence over the years, reaching 27.1% in 2022, with an overall prevalence of 35.0%, consistent with data from the ISS [21]. Glycopeptides, linezolid, daptomycin, and fifth-generation cephalosporins remain valid therapeutic options [10]. These last are usually not tested because, according to EUCAST criteria [12], methicillin-susceptible isolates can be reported as susceptible to ceftaroline and ceftobiprole without further testing. We also observed a reduction in resistance to trimethoprim-sulfamethoxazole, which could be considered a therapeutic option for treating non-severe skin and skin structure infections, where *S. aureus* is the primary isolate, as observed in our study. A German meta-analysis reported a declining trend in MRSA proportions between 2014 and 2020 while noting an increase in VRE prevalence, especially in BSIs. Conversely, they observed high resistance rates in *P. aeruginosa* against carbapenems [22]. A Spanish study showed that vancomycin resistance rates in *E. faecium* have remained low, while MRSA prevalence decreased slightly from 29.4% to 25.3% between 2001 and 2017 [23]. However, our data showed a low prevalence of VRE in 2018 and 2019, which rapidly increased in the following years. The present analysis indicates a worrying prevalence of carbapenem resistance in *K. pneumoniae* (55.0%), consistent with ISS data, which reported a prevalence of 58.4% for Sicily in 2023 [21], and consistent with RETE MIC Sicily data [24]. Although no significant trend was noted, we observed increased resistance to meropenem, meropenem-vaborbactam, and ceftazidime-avibactam. A carbapenem-sparing antimicrobial stewardship is essential to reverse the trend of carbapenem resistance for this isolate. The pathogenic role of *A. baumannii* outside of blood cultures is challenging to evaluate, as the bacterium is often considered a colonizer [25]. However, very few antibiotics are available when clinicians decide to treat the infection. Our analysis revealed a very high prevalence of resistance to all tested agents except colistin. However, colistin is limited by significant renal toxicity and unfavorable pharmacokinetic properties, especially for respiratory tract infections, where it does not reach adequate pulmonary concentrations. Cefiderocol represents an option for treating *A. baumannii* infections, although clinical data are lacking, and susceptibility testing presented technical issues, partly due to variable iron concentrations in media [10]. In our study, cefiderocol susceptibility testing was available for only 37 *A. baumannii* isolates, and resistance was detected in only 1. New antibiotics, such as the durlobactam-sulbactam combination [26] and eravacycline [27], are being studied and represent promising options for treating *A. baumannii* infections. For *P. aeruginosa*, the resistance profile appears less concerning. Our analysis shows a reduction in resistance to carbapenems and ciprofloxacin, as reported by ISS [21]. Moreover, ceftolozane-tazobactam offers a valid carbapenem-sparing option, crucial for managing difficult-to-treat strains [25], with resistance rates currently around 5% in Italy [21]. Finally, *Enterobacter* spp. was the least prevalent among ESKAPE pathogens, showing low levels of resistance to carbapenems and a decreasing trend in cefepime resistance. *Enterobacter* spp. belongs to a group of organisms, including *Citrobacter freundii*, *Serratia marcescens*, *Morganella morganii*, and *Providencia* spp., characterized by inducible AmpC β-lactamase production [25]. Consequently, cefepime remains the first therapeutic choice, especially for severe infections, regardless of susceptibility to penicillins or third-generation cephalosporins [25]. We also observed a reduction in ciprofloxacin resistance, which can be considered a β-lactam-sparing option. In our analysis, patients with positive blood cultures for ESKAPE bacteria showed high mortality rates of 38.1%. We found that *A. baumannii* and *K. pneumoniae* were associated with higher mortality in patients with bacteremia. Additionally, we noted higher resistance prevalence to ceftazidime, piperacillin-tazobactam, and meropenem in *K. pneumoniae* isolates from blood cultures compared to other specimens. A Greek study on patients with BSIs caused by ESKAPE pathogens reported increasing mortality associated with drug resistance. MDR was lowest for *P. aeruginosa* (30%) and highest for *A. baumannii* (97%), with inpatient mortality rates of 22%, 35%, and 63% for non-MDR, MDR, and XDR isolates, respectively [28]. A previous Italian study evaluating incidence and antimicrobial resistance trends in BSIs caused by ESKAPE between 2009 and 2015 showed a significant increase in hospital-onset *K. pneumoniae* BSIs. Increasing carbapenem resistance was reported for *K. pneumoniae*, while a decreasing trend in resistance to carbapenems, ceftazidime, and ciprofloxacin was observed for *P. aeruginosa*. MRSA isolates increased during the study period [29]. Another recent study at a major hospital in Southern Italy showed that ESKAPE bacteria constituted 38.7% of species isolated from positive blood cultures from January 2020 to December 2022, with *S. aureus* being the main BSI pathogen (26.3%), followed by *K. pneumoniae* (15.8%). Significant resistance rates were found, including 35% of *S. aureus* resistant to oxacillin and over 90% of *A. baumannii* resistant to carbapenems [30]. Our data, however, showed a higher prevalence of *K. pneumoniae* in blood cultures (32%), followed by *S. aureus* (20%). Similar MRSA and VRE prevalences were observed during the same period, along with carbapenem resistance for *A. baumannii*. As in our data, the lowest resistance level was observed for colistin. Unlike our findings, a significant reduction in resistance to meropenem was noted for *K. pneumoniae*, while it increased for *P. aeruginosa*. Finally, our study has limitations; antimicrobial resistance data were not available for 100% of the isolates, and resistance profile data for new antibiotics such as ceftazidime-avibactam, meropenem-vaborbactam, cefiderocol, and fifth-generation cephalosporins were scarce. Additionally, the same isolate may be repeated if obtained more than 7 days apart from the previous one.

## 5. Conclusions

At the University Hospital of Palermo, a concerning prevalence of carbapenem resistance in *K. pneumoniae* isolates and vancomycin resistance in *E. faecium* was highlighted. *A. baumannii* demonstrated persistently elevated levels of resistance to all tested antibiotics except for colistin over the course of the study. Conversely, *P. aeruginosa* and *S. aureus* exhibited a decline in carbapenem and oxacillin resistance, respectively. Additionally, our study highlighted higher resistance levels to meropenem, ceftazidime, and piperacillin-tazobactam in *K. pneumoniae* blood isolates compared to those from other sample types, and it revealed higher mortality prevalence among patients with bacteremia caused by *A. baumannii* and *K. pneumoniae*. Local epidemiological studies are instrumental in evaluating the prevalence of antibiotic resistance, guiding rational and empirical antibiotic therapy, and implementing measures to curb the development of antibiotic resistance through antimicrobial stewardship programs.

## Figures and Tables

**Table 1:** Absolute frequencies of isolates over the years and antimicrobial resistance prevalence from 2018 to 2023.

Prevalence of Resistance (Resistant Isolates/Available Antibiotic Sensitivity Testing) According to Year						
Bacteria and Antibiotics	2018	2019	2020	2021	2022	2023
*E. faecium*	*n* = 99	*n* = 112	*n* = 133	*n* = 246	*n* = 251	*n* = 101
Ampicillin ^1^	0	0	0	0	45/54 (83.3%)	71/99 (71.7%)
Vancomycin	4/94 (4.3%)	1/111 (0.9%)	23/133 (17.3%)	55/242 (22.7%)	73/235 (31.1%)	22/100 (22.0%)
Teicoplanin	6/94 (6.4%)	0/111 (0.0%)	23/133 (17.3%)	55/244 (22.5%)	74/251 (29.5%)	22/100 (22.0%)
Gentamicin	18/58 (61.7%)	61/111 (54.9%)	89/130 (68.5%)	146/244 (59.8%)	167/251 (66.5%)	40/100 (40.0%)
Linezolid	0/94 (0.0%)	3/111 (2.7%)	0/133 (0.0%)	0/244 (0.0%)	1/250 (0.4%)	2/99 (2.0%)
Imipenem	79/91(86.8%)	90/108 (83.3%)	122/132 (92.4%)	233/244 (95.5%)	219/234 (93.6%)	80/101 (79.2%)
*S. aureus*	*n* = 244	*n* = 318	*n* = 274	*n* = 325	*n* = 371	*n* = 210
Oxacillin	99/238 (41.2%)	114/300 (38.0%)	101/273 (37.0%)	113/317 (35.6%)	59/218 (27.1%)	57/203 (28.1%)
Vancomycin	1/239 (0.4%)	5/301 (1.7%)	2/274 (0.7%)	1/317 (0.3%)	0/218 (0.0%)	4/203 (2.0%)
Teicoplanin	2/239 (0.8%)	3/300 (1.0%)	2/273 (0.7%)	5/317 (1.6%)	0/218 (0.0%)	3/208 (1.4%)
Gentamicin	60/239 (25.1%)	55/301 (18.3%)	42/273 (15.4%)	63/317 (19.9%)	21/218 (9.6%)	29/208 (13.9%)
Linezolid	0/238 (0.0%)	0/300 (0.0%)	0/274 (0.0%)	2/317 (0.6%)	1/218 (0.4%)	5/208 (2.4%)
Daptomycin	0/229 (0.0%)	0/299 (0.0%)	4/274 (1.5%)	18/317 (5.7%)	2/218 (0.9%)	3/208 (1.4%)
TMP-SMX	29/238 (12.2%)	17/301 (5.6%)	17/273 (6.2%)	16/316 (5.1%)	6/218 (2.7%)	4/206 (1.9%)
Clindamycin	99/238 (41.6%)	113/299 (37.8%)	107/272 (39.3%)	134/317 (42.3%)	89/218 (40.8%)	89/206 (43.2%)
Tetracycline	21/239 (8.8%)	24/301 (8.0%)	42/273 (15.4%)	27/317 (8.5%)	21/218 (9.6%)	31/208 (14.9%)
Moxifloxacin	99/228 (43.4%)	85/299 (28.4%)	87/272 (32.0%)	115/314 (36.6%)	65/218 (29.8%)	42/201(20.9%)
*K. pneumoniae*	*n* = 510	*n* = 559	*n* = 585	*n* = 966	*n* = 795	*n* = 397 (%)
Amox-CA	426/502 (84.9%)	452/546 (82.8%)	480/583 (82.3%)	769/916 (84.0%)	524/676 (77.5%)	287/397 (72.3%)
Ceftazidime	394/504 (78.2%)	437/544 (80.3%)	462/583 (79.2%)	759/916 (82.9%)	516/676 (76.3%)	287/397 (72.3%)
Pip-taz	371/505 (73.5%)	409/544 (75.2%)	425/583 (72.9%)	701/916 (76.5%)	463/676 (68.5%)	255/387 (65.9%)
Cefepime	387/503 (76.9%)	435/544 (80.0%)	459/583 (78.7%)	754/915 (82.4%)	503/674 (74.6%)	286/397 (72.0%)
Cefto-taz	0	0	32/46 (69.6%)	651/710 (91.7%)	377/601 (62.7%)	213/388 (54.9%)
Meropenem	240/502 (47.8%)	268/544 (49.3%)	317/581(54.6%)	610/913 (66.8%)	363/674 (53.8%)	181/386 (46.9%)
Imipenem	196/499 (39.3%)	237/542 (43.7%)	336/577 (58.2%)	621/913 (68.0%)	379/676 (56.1%)	184/397 (46.3%)
Ertapenem	334/501 (66.7%)	367/544 (67.5%)	404/582 (69.4%)	679/915 (74.2%)	414/676 (61.2%)	212/397 (53.4%)
CAZ-AVI	0	0	4/104 (3.8%)	98/714 (13.7%)	40/617 (6.5%)	32/382 (8.4%)
Mer-Vab	0	0	0	1/152 (0.7%)	13/475 (2.7%)	21/355 (5.9%)
TMP-SMX	284/505 (56.2%)	243/546 (44.5%)	342/582 (58.8%)	442/916 (48.3%)	289/676(42.7%)	170/387 (43.9%)
Ciprofloxacin	394/504 (78.2%)	442/544 (81.3%)	458/582 (78.7%)	741/915 (81.0%)	483/675 (71.5%)	264/397 (66.5%)
Gentamicin	300/505 (59.4%)	277/546 (50.7%)	332/583 (56.9%)	634/909 (69.7%)	387/676 (57.2%)	287/397 (72.3%)
Colistin	23/429 (5.4%)	47/407 (10.3%)	42/439 (9.6%)	78/878 (8.9%)	53/650 (8.1%)	19/378 (5.0%)
*A. baumannii*	*n* = 131	*n* = 158	*n* = 275	*n* = 412	*n* = 424	*n* = 215
Meropenem	124/130 (95.4%)	139/155 (89.7%)	264/274 (96.4%)	374/399 (93.7%)	408/423 (96.5%)	209/213 (98.1%)
Imipenem	123/129 (95.3%)	142/157 (90.4%)	265/273 (97.1%)	375/399 (94.0%)	408/423 (96.5%)	210/214 (98.1%)
TMP-SMX	123/130 (94.6%)	142/158 (89.9%)	200/274 (73.0%)	283/399 (70.9%)	362/423 (85.6%)	201/215 (93.5%)
Ciprofloxacin	125/129 (96.9%)	149/158 (94.3%)	266/274 (97.1%)	362/398 (91.0%)	398/423 (94.1%)	209/215 (97.2%)
Gentamicin	127/130 (97.7%)	146/158 (92.4%)	261/273 (95.6%)	354/398 (88.9%)	364/423 (86.1%)	172/214 (80.4%)
Colistin	2/129 (1.6%)	6/154 (3.9%)	4/264 (1.5%)	6/392 (1.5%)	7/374 (1.9%)	5/208 (2.4%)
*P. aeruginosa*	*n* = 318	*n* = 386	*n* = 450	*n* = 637	*n* = 637	*n* = 332
Ceftazidime	96/317 (30.3%)	78/375 (20.8%)	89/448 (19.9%)	155/535 (29.0%)	142/563 (25.2%)	93/324 (28.7%)
Pip-taz	99/315 (31.4%)	88/377 (23.3%)	89/449 (19.8%)	138/535 (25.8%)	131/564 (23.2%)	83/323 (25.7%)
Cefepime	90/309 (29.1%)	90/368 (24.5%)	95/446 (21.3%)	149/535 (27.9%)	126/561 (22.4%)	78/329 (23.7%)
Cefto-taz	0	0	0/7 (0.0%)	0/267 (0.0%)	23/456 (5.0%)	16/309 (5.2%)
Meropenem	86/317 (27.1%)	86/372 (23.1%)	85/449 (18.9%)	108/533 (20.3%)	105/564 (18.6%)	50/318 (15.7%)
Imipenem	108/309 (35.0%)	102/371(27.5%)	120/446 (26.9%)	162/533 (30.4%)	151/563 (26.8%)	78/326 (23.9%)
CAZ-AVI	0	0	0/8 (0.0%)	31/308 (10.1%)	34/495 (6.9%)	15/307 (4.9%)
Ciprofloxacin	116/317 (36.6%)	117/376 (31.1%)	123/448 (27.5%)	153/535 (28.6%)	126/564 (22.3%)	73/332 (22.0%)
Tobramycin	73/316 (23.1%)	62/376 (16.5%)	74/448 (16.5%)	50/534 (9.4%)	52/562 (9.2%)	30/323 (9.3%)
Colistin	2/273 (0.7%)	4/308 (1.3%)	3/375 (0.8%)	5/510 (1.0%)	11/542 (2.0%)	3/299 (1.0%)
*Enterobacter* spp.	*n* = 150	*n* = 120	*n* = 122	*n* = 194	*n* = 144	*n* = 93
Ciprofloxacin	49/133 (36.8%)	29/96 (30.2%)	30/121 (24.8%)	35/189 (18.5%)	13/144 (9.0%)	13/93 (14.0%)
Colistin	2/100 (2.0%)	3/68 (4.4%)	1/90 (1.1%)	4/172 (2.3%)	5/121 (4.1%)	0/81 (0.0%)
Cefepime	47/133 (35.3%)	26/95 (27.4%)	29/122 (23.8%)	43/189 (22.8%)	21/131 (16.0%)	12/93 (12.9%)
Amox-CA	130/132 (98.5%)	96/96 (100.0%)	122/122 (100.0%)	188/189 (99.5%)	130/132 (98.5%)	91/93 (97.8%)
Gentamicin	28/133 (21.1%)	20/96 (20.8%)	22/122 (18.0%)	34/189 (18.0%)	17/132 (12.9%)	6/93 (6.5%)
Ertapenem	34/132 (25.8%)	23/93 (24.7%)	32/122 (26.2%)	46/189 (24.3%)	21/132 (15.9%)	14/93 (15.1%)
Imipenem	7/133 (5.3%)	7/96 (7.3%)	9/121 (7.4%)	11/188 (5.9%)	7/132 (5.3%)	5/93 (5.4%)
Meropenem	9/131 (6.9%)	4/95 (4.2%)	7/122 (5.7%)	9/186 (4.8%)	2/132 (1.5%)	4/90 (4.4%)
TMP-SMX	47/133 (35.3%)	25/96 (26.0%)	36/122 (29.5%)	55/189 (29.1%)	11/132 (8.3%)	17/90 (18.9%)
Ceftazidime	53/133 (39.8%)	33/95 (34.7%)	45/122 (36.9%)	70/189 (37.0%)	40/132 (30.3%)	24/93 (25.8%)
CAZ-AVI	0	0	1/7 (14.3%)	11/99 (11.1%)	2/111 (1.8%)	4/82 (4.9%)
Cefto-taz	0	0	2/7 (28.6%)	17/100 (17.0%)	2/121 (1.6%)	0/84 (0.0%)
Mer-vab	0	0	0	0/4 (0.0%)	0/55 (0.0%)	1/56 (1.8%)
Pip-taz	47/133 (35.3%)	31/95 (32.6%)	39/122 (32.0%)	47/189 (24.9%)	37/132 (28.0%)	19/90 (21.1%)

^1^ Not routinely tested for *E. faecium*; Amox-CA: amoxicillin-clavulanic acid; Pip-taz: piperacillin-tazobactam; Cefto-taz: ceftolozane-tazobactam; CAZ-AVI: ceftazidime-avibactam; Mer-vab: meropenem-vaborbactam; TMP-SMX: trimethoprim-sulfamethoxazole.

**Table 2:** Multivariable analysis of mortality upon admission in patients with ESKAPE isolates in blood cultures.

Variables	AOR	CI	*p* Value
ICU admission	5.15219	3.96966–6.68698	<0.001
CCI	1.07224	1.00969–1.13867	0.023
*K. pneumoniae*	1.68457	1.28542–2.20768	<0.001
*A. baumannii*	2.00344	1.44249–2.78252	<0.001

ICU: intensive care unit, CCI: Charlson comorbidity index.

**Table 3:** Comparison of antimicrobial resistance prevalence between blood isolates and isolates from other specimens.

Bacteria and Antibiotics	Blood Culture	Other Specimen	*p*
*E. faecium*			
Vancomycin	42/184 (22.8%)	136/731 (18.6%)	0.196
*S. aureus*			
Oxacillin	111/325 (34.1%)	432/1224 (35.3%)	0.701
Vancomycin	3/326 (0.9%)	10/1226 (0.8%)	0.853
Daptomycin	5/324 (1.5%)	22/1221 (1.8%)	0.752
Linezolid	0/325 (0%)	6/1230 (0.5%)	0.207
*K. pneumoniae*			
Ceftazidime	434/515 (84.3%)	2421/3105 (78.0%)	0.001
Pip-taz	410/511 (80.2%)	2214/3100 (71.4%)	<0.001
Cefepime	426/596 (71.5%)	2398/3020 (79.4%)	<0.001
Meropenem	336/508 (66.1%)	1643/3092 (53.1%)	<0.001
CAZ-AVI	23/327 (7.0%)	114/1490 (7.6%)	0.701
Ciprofloxacin	419/513 (81.7%)	2363/3104 (76.1%)	0.006
*A. baumannii*			
Colistin	3/298 (1.0%)	27/1223 (2.2%)	0.181
*P. aeruginosa*			
Ceftazidime	44/190 (23.1%)	609/2372 (25.7%)	0.715
Pip-taz	35/189 (18.5%)	593/2374 (25.0%)	0.047
Cefto-taz	0/93 (0%)	39/946 (4.1%)	0.046
Cefepime	38/188 (20.2%)	590/2360 (25.0%)	0.142
Meropenem	45/187 (24.1%)	475/2366 (20.1%)	0.192
CAZ-AVI	3/101 (3.0%)	77/1017 (7.6%)	0.087
Ciprofloxacin	42/190 (22.1%)	666/2382 (27.9%)	0.082
*Enterobacter* spp.			
Ciprofloxacin	25/112 (22.3%)	144/664 (21.7%)	0.880
Cefepime	29/115 (25.2%)	149/648 (23.0%)	0.422
Meropenem	6/115 (5.2%)	29/641 (4.5%)	0.744

Pip-taz: piperacillin-tazobactam, Cefto-taz: ceftolozane-tazobactam, CAZ-AVI: ceftazidime-avibactam.

## Data Availability

Data will be made available upon request.

## Abbreviations

The following abbreviations are used in this manuscript:

AMR	Antimicrobial resistance
ESKAPE	*Enterococcus faecium*, *Staphylococcus aureus*, *Klebsiella pneumoniae*, *Acinetobacter baumannii*, *Pseudomonas aeruginosa*, and *Enterobacter* spp.
ICU	Intensive care unit
WHO	World Health Organization
MDR	Multidrug-resistant
BSI	Bloodstream infection
EUCAST	European Committee on Antimicrobial Susceptibility Testing
AST	Antimicrobial susceptibility testing
H-SDF	Hospital discharge form
IQR	Interquartile range
cOR	Crude odds ratio
aOR	Adjusted odds ratio
VRE	Vancomycin-resistant *E. faecium*
MRSA	Methicillin-resistant *S. aureus*
